# Clinical Performance and Immediate Child and Parental Satisfaction with BioFlx Crowns in Primary Molars: A Prospective, Single-Arm, Non-Randomized Interventional Study

**DOI:** 10.3390/children13091258

**Published:** 2026-09-16

**Authors:** Shahad N. Abudawood, Ahmed Essam, Lolo A. Almansour, Wed M. Kassar, Sara M. Bagher, Osama M. Felemban

**Affiliations:** 1 Pediatric Dentistry Department, Faculty of Dentistry, King Abdulaziz University, Jeddah 21589, Saudi Arabia; sbagher@kau.edu.sa (S.M.B.); omfelemban@kau.edu.sa (O.M.F.); 2 Senior Registrar Pediatric Dentist, King Abdulaziz University Dental Hospital, Jeddah 21589, Saudi Arabia; 3 Training Resident-Pediatric Dentistry, Pediatric Dentistry Department, Faculty of Dentistry, King Abdulaziz University, Jeddah 21589, Saudi Arabia

**Keywords:** BioFlx, full-coverage restoration, pediatric dentistry, primary molars, satisfaction

## Abstract

**Background/Objectives:** BioFlx crowns have recently been introduced as an aesthetic full-coverage option for restoring primary molars; however, clinical evidence remains limited. This prospective, single-arm, non-randomized interventional study evaluated the short-term clinical performance of BioFlx crowns in primary molars over six months and assessed immediate satisfaction of children and their parents after crown placement. **Methods:** Healthy children aged 6–9 years or children with mild, stable systemic medical conditions who were cooperative and required at least one full-coverage restoration in a primary molar were recruited from the pediatric dentistry clinics at King Abdulaziz University Faculty of Dentistry. BioFlx crowns were placed by trained pediatric dentistry residents under a consultant’s supervision. Immediately after crown cementation, proximal contact and occlusion were recorded. At three- and six-month follow-up visits, clinical outcomes evaluated were the proximal contacts, occlusion, crown retention, staining, wear of the opposing tooth, marginal integrity, and any changes in the crown material surface. In addition, plaque accumulation and gingival health around each crowned tooth were assessed at the tooth level. Immediate post-treatment satisfaction of children and their parents was assessed following crown cementation. **Results:** Twenty-eight children received 71 BioFlx crowns. The gingival health was favorable, with most teeth showing no bleeding throughout the follow-up visits. At six months, all evaluated crowns maintained ideal occlusion and showed no opposing-tooth wear; 98.4% remained retentive, 91.8% showed no staining, 93.4% maintained ideal marginal integrity, and minor surface indentations were common at 59%, while perforations were limited to 4.9% at six-month assessment. Children and parents reported high overall satisfaction with appearance (9.8 ± 0.4 and 9.5 ± 0.8, respectively). **Conclusions:** Within the selected cohort, BioFlx crowns demonstrated favorable short-term clinical performance over six months and high immediate post-treatment satisfaction among children and parents; however, surface indentations were frequently observed, and occasional clinically unacceptable perforations were noted.

## 1. Introduction

Pediatric dentists have always faced the challenge of satisfactorily restoring primary teeth, improving aesthetics, and managing space and function. For many years, Stainless-Steel Crowns (SSCs) have been used to treat multi-surface carious lesions affecting primary molars [1,2].

Stainless-steel crowns are inexpensive, easy to place, and practical, with a high success rate in restoring primary molars, and are considered the standard of care. However, one main drawback of SSCs is their non-aesthetic metal appearance and color mismatch [3]. A recent study was conducted among children aged 6–10 years, and a considerable number reported aesthetic concerns, negative social experiences, psychosocial discomfort, and reduced self-confidence related to the appearance of SSCs. Approximately 39% of the children experienced bullying, most commonly verbal teasing. In addition, satisfaction with the crown’s appearance, whether the shape and/or color, was relatively low [4].

Children may become increasingly aware of their appearance and participate more actively in treatment-related decisions as they mature, while parents and children may also express greater interest in aesthetic treatment options for discolored or compromised teeth [5,6]. Modern dental practice has shifted towards child-centered care and shared parent–dentist decision-making, in which parents play an important role in dental treatment planning of pediatric patients. It is imperative to discuss treatment options available to parents prior to treatment, including advantages, disadvantages, and alternatives [4], as parental satisfaction plays a crucial role in healthcare, particularly regarding children’s oral health [7].

Therefore, new aesthetic alternatives, such as prefabricated zirconia crowns (PZCs), have been developed. These crowns offer more aesthetically pleasing alternatives to traditional SSCs, which some parents may reject because of their non-aesthetic, metallic appearance despite their high clinical success [8]. PZCs generally require greater tooth reduction, rely on passive rather than crimped adaptation, are more technique-sensitive during preparation and cementation, and are relatively more expensive [3]. These limitations have encouraged the development of alternative aesthetic full-coverage restorations that aim to combine acceptable clinical performance with improved appearance and simplified clinical handling. BioFlx^®^ crowns (NuSmile, Houston, TX, USA) might offer such an option. What makes BioFlx crowns appealing to pediatric dentists is that their tooth preparation, size selection, fitting, and cementation techniques are similar to those of SSCs. BioFlx crowns were newly introduced into the market in 2023 and, according to the manufacturer, are fabricated from biocompatible, high-impact hybrid resin and are free from both metal and bisphenol A-glycidyl methacrylate (Bis-GMA). They are supplied as pre-contoured and pre-crimped, as in SSCs [9,10]. Although BioFlx crowns offer potential advantages in aesthetics and clinical handling, they are relatively new to pediatric restorative dentistry, and clinical evidence regarding their retention, material integrity, gingival response, durability, and patient satisfaction remains limited. Further prospective studies are needed to evaluate their clinical performance relative to established full-coverage crown options.

Therefore, the aim of this prospective, single-arm, non-randomized interventional study was to assess the clinical performance of BioFlx crowns as a full-coverage restorative treatment option in primary molars in pediatric patients evaluated at a six-month follow-up. Also, the immediate satisfaction of the children and parents was recorded.

## 2. Materials and Methods

### 2.1. Ethical Approval and Study Registration

Ethical approval was obtained from the Research Ethics Committee at the Faculty of Dentistry, King Abdulaziz University, Jeddah, Saudi Arabia (53-03-24). This trial was retrospectively registered at ClinicalTrials.gov (Identifier: NCT07471360).

### 2.2. Study Design, Setting, and Participant Recruitment

This prospective, single-arm, non-randomized interventional study was conducted at the pediatric dentistry clinics at King Abdulaziz University Faculty of Dentistry (KAUFD) in Jeddah, Saudi Arabia, between December 2024 and January 2026.

### 2.3. Prespecified Study Outcomes

The prespecified clinical performance outcomes of BioFlx crowns included plaque accumulation, gingival health, proximal contacts, occlusion, crown retention, staining, wear of the opposing tooth, marginal integrity, and surface integrity of the crown material.

Immediate post-treatment satisfaction among children and their parents was also prespecified as a study outcome and was assessed following crown placement. These outcomes and the scheduled follow-up assessments were documented in the ethics-approved study protocol before participant recruitment.

The prespecified study outcomes included clinical and radiographic assessments at the scheduled follow-up visits. The original ethical approval and registry included follow-up assessments up to 24 months. However, a technical failure of the institutional radiographic data-storage system resulted in the loss of a substantial proportion of the follow-up radiographs, precluding a complete and reliable radiographic analysis. Consequently, radiographic outcomes were not included in the present analysis. The current manuscript reports the available clinical outcomes through the six-month follow-up period. The 24-month outcomes will be analyzed and reported separately after completion of the scheduled follow-up period.

### 2.4. Inclusion and Exclusion Criteria

The inclusion criteria were applied at both the participant and primary molar levels. At the participant level, children aged six to nine years were included if they met the following criteria: (1) healthy or with mild, stable systemic medical conditions such as controlled asthma, with American Society of Anesthesiologists (ASA) Classification I or II status [11]; (2) cooperative in dental treatment with a rating of “definitely positive” or “positive” on the Frankl Behavioral Rating Scale [12]; (3) not known to be allergic to any components of the dental materials used in the study (20% benzocaine topical gel (Sky-Caine^®^ Gel, Skydent Inc., Manhattan, NY, USA), local anesthesia using 2% Mepivacaine containing 1:100,000 epinephrine (Scandonest^®^ 2% Special, Septodont, Saint-Maur-des-Fossés, France), Type I glass ionomer luting cement (Ketac™ Cem Radiopaque, 3M, Neuss, Germany) and BioFlx^®^ crowns (NuSmile, Houston, TX, USA)). They also had at least one carious primary molar requiring a full-coverage restoration, as determined by clinical and radiographic inclusion criteria for primary molars and assessed by at least two trained and calibrated pediatric consultants. The clinical and radiographic inclusion criteria at the primary molar level included primary molars that are fully erupted in occlusion and functional, with at least one proximal contact with an adjacent tooth, vital or requiring vital pulp therapy, no clinical or radiographic signs or symptoms of periradicular pathology and exhibiting normal interproximal bone levels with no more than one-third root resorption radiographically.

Children were excluded if they presented with severe malocclusion that could compromise crown placement or evaluation of the clinical outcomes, including severe crowding or displacement of the study tooth, severe open bite, or crossbite involving the study tooth. Children diagnosed with active periodontal disease evidenced by clinical attachment loss and/or radiographic alveolar bone loss, and those requiring comprehensive dental treatment under general anesthesia were also excluded.

Potentially eligible children who met the inclusion criteria at the participant level during their initial examination or routine follow-up visit were screened for the clinical and radiographic inclusion criteria at the primary molar level after obtaining parental/guardian screening consent and providing assent from children aged seven years and older.

For the radiographic evaluation, standardized baseline bitewing radiographs (BWs) were obtained as part of their initial examination or regular follow-up appointment, and no additional radiographs were obtained for research purposes. In addition, the clinical examination and radiographic evaluation were performed independently by at least two trained and calibrated pediatric consultants. If the two pediatric consultants disagreed, a third trained and calibrated pediatric consultant performed the clinical and radiographic examination.

### 2.5. Informed Consent and Child Assent

Children who met the inclusion criteria at the participant and primary molar levels were considered eligible. Parental/guardian enrollment consent was obtained after a detailed explanation of the treatment, including possible outcomes, risks, benefits, and discomforts, and was provided in accordance with the Declaration of Helsinki, and assent forms were obtained from participating children aged seven years and older. A treatment appointment was scheduled for them.

### 2.6. Sociodemographic Data Collection

At the scheduled appointment, the participating children and their parents’/guardians’ demographic data (age, gender, type of school, number of siblings, child’s order of birth in the family, parental age, parental level of education, occupation, and the average monthly family income in Saudi Riyals) were recorded. The classification of average monthly family income was based on central statistics and information, with <7000 Saudi Riyals (SR) per month considered low income; 7000–10,000 SR per month considered low-to-middle income; 10,000–16,000 SR per month considered middle-to-high income; and >16,000 SR per month considered high income [13].

### 2.7. Clinical Evaluation and Follow-Up

Before preparation, participant-level baseline data were recorded, including dental history and a comprehensive preoperative full-mouth oral health assessment. This assessment included dental caries experience recorded using the dmft/DMFT indices, oral hygiene using the Greene and Vermillion Simplified Oral Hygiene Index (OHI-S), and gingival health assessed using the Löe and Silness Gingival Index (GI) [14,15]. In addition, the included teeth’s status (arch, tooth type, side, number of carious surfaces, pulp status, opposing teeth/material, occluding surfaces in contact, and adjacent tooth/teeth status) was recorded.

The OHI-S was scored after the participant chewed a disclosing tablet (Red-Cote; John O. Butler, Chicago, IL, USA) and rinsed with water, by running the side of an explorer over the buccal surface of teeth #16 or #55, #11 or #51, #26 or #65 and #31 or #71, as well as the lingual surface of teeth #36 or #75 and #46 or #85. The index was calculated by summing the plaque debris index ranging from zero to three and taking the mean. The scores of the six teeth were averaged, and subjects were categorized as good (0–0.6), fair (0.7–1.8), or poor (1.9–3.0) oral hygiene. Gingival health was evaluated using the Löe and Silness Gingival Index [15]. A periodontal probe was gently passed along the gingival sulcus of six index teeth and scored from zero to three: zero indicated clinically healthy gingiva; one indicated mild inflammation without bleeding on probing; two indicated moderate inflammation with bleeding on probing; and three indicated severe inflammation with a tendency toward spontaneous bleeding. The individual scores were averaged to obtain a subject-level gingival index score, and participants were categorized according to the severity of gingival inflammation as follows: 0.1–1.0 = mild gingivitis, 1.1–2.0 = moderate gingivitis, and 2.1–3.0 = severe gingivitis.

Furthermore, the clinical assessment comprised multiple parameters, including selected criteria adapted from the modified United States Public Health Service (USPHS) system for the direct clinical evaluation of restorations [16], namely marginal integrity and crown staining; crown-specific clinical criteria adapted from Donly et al. [17], namely proximal contact, occlusion, crown retention, and wear of the opposing tooth; established plaque accumulation [14] and bleeding on probing [15]; and a study-specific criterion for evaluating the surface integrity of BioFlx crowns, as presented in Table 1.

Immediately after cementation, the clinical assessment included proximal contact (mesial and distal), occlusion, crown retention, and marginal integrity. At the three- and six-month follow-up visits, the evaluation included the proximal contacts (mesial and distal), occlusion, crown retention after cementation, crown staining, wear of the opposing tooth, marginal integrity, and any changes in the crown material’s surface. In addition, plaque accumulation and bleeding on probing around the included crowns were recorded. An explorer was used to record any catch or gap along all margins, as well as any changes in the surface of the crown material.

All the clinical parameters were evaluated and recorded by two trained and calibrated pediatric dentistry residents. Upon disagreement, a third trained and calibrated examiner was involved.

### 2.8. Clinical Procedure and Crown Placement

**Figure 1:**
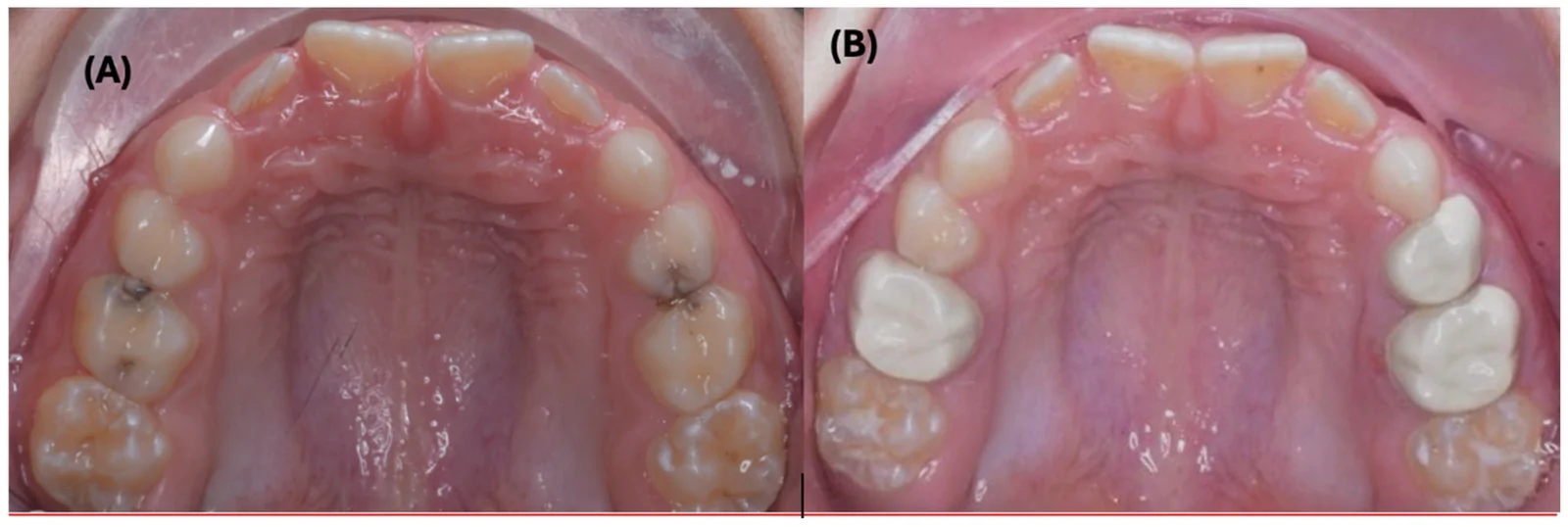
Maxillary occlusal views of a 7-year-old female patient: (**A**) preoperative view showing interproximal carious lesions involving the maxillary right second primary molar (tooth #55) and maxillary left first and second primary molars (teeth #64 and #65); (**B**) three-month postoperative appearance following restoration of the affected teeth with BioFlx crowns.

The treatment was provided by two trained pediatric dentistry residents, with an assistant, in accordance with the manufacturer’s instructions and under the supervision of a consultant pediatric dentist. A new set of burs was used to prepare each crown. Appropriate pain management was delivered using 20% benzocaine topical gel (Sky-Caine^®^ Gel, Skydent Inc., Manhattan, NY, USA), followed by local anesthesia using 2% Mepivacaine containing 1:100,000 epinephrine (Scandonest^®^ 2% Special, Septodont, Saint-Maur-des-Fossés, France). Then, preparation started under rubber dam isolation with a 1–1.5 mm occlusal reduction using a pear-shaped bur, followed by interproximal reduction with a tapered diamond bur. Complete or partial caries removal was carried out using a handpiece and/or an excavator to remove carious dentine. If vital pulp therapy was required, it was performed using NeoMTA^®^ (NuSmile, Houston, TX, USA), and the chamber was filled using GC Fuji™ II LC Capsule (GC Corporation, Tokyo, Japan). BioFlx^®^ crowns (NuSmile, Houston, TX, USA) were cemented with Type I glass ionomer luting cement (Ketac™ Cem Radiopaque, 3M, Neuss, Germany); excess cement was removed, and occlusion was verified. If the participating child was diagnosed with more than one eligible primary molar, the second eligible primary molar was prepared either on the same visit if it was in the same quadrant or in a similar manner on a subsequent visit. Crowns that showed ideal (alpha) occlusion, retention after cementation, and marginal integrity were considered acceptable and included in the study. The clinical evaluation was conducted immediately after cementation, at three- and six-month follow-up appointments. The preoperative and three-month postoperative occlusal views of a 7-year-old female patient who received BioFlx crowns in the maxillary right second, left first, and second primary molars are presented in Figure 1.

### 2.9. Child and Parental Satisfaction Assessment

After crown cementation, the immediate post-treatment satisfaction of children and their parents with BioFlx crowns was assessed using a questionnaire adapted from a previously validated Arabic questionnaire with minor modification [10]. Children and parents were interviewed individually in a separate setting and were asked to rate their satisfaction with the crown’s color, shape, size, and overall appearance. Each item was scored on a 10-point Likert scale ranging from 1 (“not satisfied at all”) to 10 (“very satisfied”), with higher scores indicating greater satisfaction. The questionnaire was selected to provide a structured assessment of satisfaction with specific aesthetic characteristics of the crowns as well as overall satisfaction immediately following treatment. As the adapted version was not independently validated in this age group, the satisfaction findings should be interpreted as exploratory.

### 2.10. Statistical Analysis

Data were analyzed using SPSS version 20.0. Categorical variables were summarized as frequencies and percentages. Continuous satisfaction scores were summarized as mean ± standard deviation (SD). The statistical unit was the child for demographic characteristics and satisfaction outcomes, and the crown/tooth for clinical performance outcomes.

## 3. Results

A total of 28 children were included in the study, and 71 crowns were placed. All 71 crowns were evaluated at baseline and at the three-month follow-up. At the six-month follow-up, 22 participants with 61 crowns were available for evaluation because six participants with 10 crowns did not attend the scheduled recall visit despite attempts to contact them.

The demographic characteristics and the oral health status of the participants are presented in Table 2. Most children were aged 8–9 years (57.1%), with a slightly higher proportion of females (57.1%) than males. More than half attended public schools (57.1%), and the majority had two or more siblings (57.1%), with over half being first-borns. All fathers and nearly two-thirds of the mothers (64.3%) were employed. The family average monthly income predominantly fell within the middle-to-high income category (67.9%). Overall, the cohort represents a relatively homogeneous, well-educated population suitable for clinical evaluation of BioFlx crowns.

Furthermore, slightly more than half of them (53.6%) had no prior dental history. The majority exhibited a high level of caries experience, with 71.4% having a dmft/DMFT score between six and 10. Most participants (53.6%) had fair oral hygiene, and only a small proportion (3.6%) had good oral hygiene, according to the OHI-S. Also, most participants had mild gingivitis (96.4%).

Most crowns were placed in the maxillary arch (59.2%), with a higher proportion involving first primary molars (57.7%). The majority of teeth presented with caries affecting two surfaces (87.3%). Most teeth were vital with normal pulp status (91.5%). Opposing and adjacent teeth were predominantly natural, indicating favorable occlusal and proximal conditions for crown placement. Overall, the teeth selected for treatment were largely structurally intact, with limited pulpal involvement, supporting their suitability for full-coverage restorations with BioFlx crowns. Table 3 describes the baseline characteristics of the primary molars prior to BioFlx crown placement.

All participants attended the three-month follow-up visit, whereas only 22 participants, with a total of 61 teeth, attended the six-month follow-up visit. With respect to plaque accumulation on the crown, about two-thirds of the crowns at three months (66.2%) and one-third of the crowns at the six-month visit (32.8%) had no debris or stains present. Although a slight increase in plaque scores was observed at six months, most teeth remained within clinically acceptable ranges, indicating adequate plaque control around BioFlx crowns over time. Gingival health outcomes were similarly favorable, with the majority of crowned teeth exhibiting no bleeding on probing at the three- and six-month follow-up visits. Proximal contact assessment showed high rates of clinically ideal contacts on both mesial and distal surfaces at all evaluation intervals, with only a small number of teeth demonstrating minor, clinically acceptable deviations. Occlusal assessment revealed that all BioFlx crowns were in ideal occlusal harmony at the follow-ups, with no cases requiring occlusal adjustment or crown replacement. Crown retention after cementation results were excellent, with all crowns remaining retentive at three months and only one crown exhibiting mobility at six-month follow-up. No complete crown debonding or loss was reported. BioFlx crown staining was minimal, limited to mild discoloration, and remained clinically acceptable and amenable to polishing. Importantly, no wear of the opposing dentition was detected at either follow-up interval. Marginal integrity was largely maintained throughout the follow-up periods, with the majority of BioFlx crowns rated as clinically ideal and a small proportion presenting minor, non-critical marginal discrepancies.

**Figure 2:**
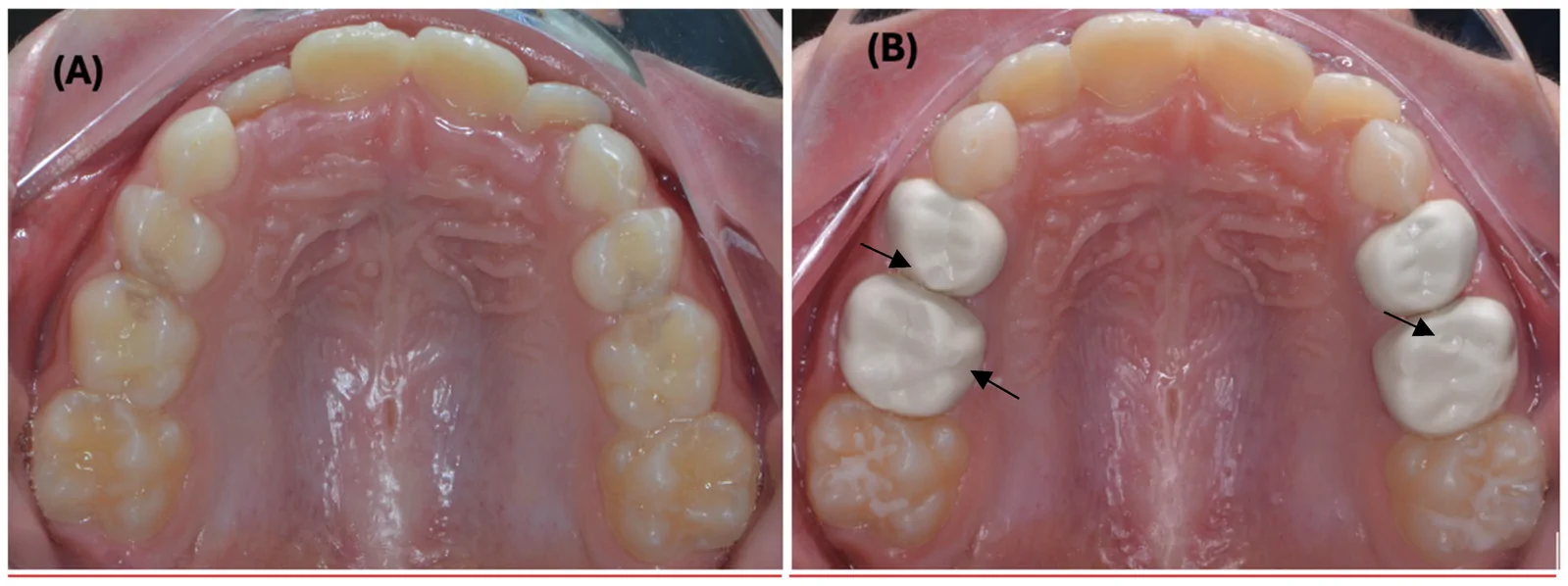
Maxillary occlusal views of an 8-year-old male patient: (**A**) preoperative clinical appearance showing previously diagnosed bilateral interproximal caries in maxillary first and second primary molars (teeth #54, #55, #64, and #65); (**B**) three-month postoperative appearance following placement of BioFlx crowns. Black arrows indicate localized occlusal surface indentations (dimples on teeth #55, #54, and #65).

Minor surface indentations (1 = Bravo) represented the most observed change and were considered clinically acceptable, while only a small number of crowns exhibited perforation (2 = Charlie) and were classified as clinically unacceptable at three months. A slight deterioration in surface integrity was observed over time, as ideal surface ratings decreased, and clinically unacceptable ratings increased at six-month assessment. Most indentations (Bravo) and perforations (Charlie) appeared at the occlusal surfaces (dimensions of the indentation/perforation were not measured in this study), and if perforation occurred, the underlying restorative material and/or dentin was exposed and considered a failure necessitating replacement with either a new BioFlx crown or SSCs based on clinical judgment and parents’ preference. Collectively, the crowns demonstrated favorable short-term clinical performance across most evaluated parameters. However, the integrity of the crown surface showed less favorable outcomes, with frequent clinically acceptable surface indentations and a small number of clinically unacceptable perforations observed during the follow-up period (Table 4). The preoperative and three-month postoperative occlusal views of an 8-year-old male patient who received BioFlx crowns in the maxillary first and second primary molars with occlusal surface indentations during the three-month follow-up are presented in Figure 2.

Table 5 presents immediate post-treatment satisfaction of children and their parents with the aesthetic outcomes of BioFlx crowns, with consistently high satisfaction scores across all evaluated parameters (mean ratings exceeding nine out of 10 on the scale).

## 4. Discussion

The findings of this study indicate that BioFlx crowns consistently demonstrated favorable clinical performance across all clinical parameters during the three- and six-month follow-up periods. Furthermore, high satisfaction among parents and children with BioFlx crowns was reported.

Our results are consistent with a recent study by Abdelhafez and Dhar (2025) [18]. In their study, they evaluated three types of pediatric crowns, including SSCs, PZCs, and BioFlx crowns, and found minimal plaque accumulation and minimal gingival inflammation around all crown types at the six-month follow-up visit, which increased at 12 months, with no statistically significant differences between the crown types. In addition, they reported that all crown types showed acceptable clinical performance at both follow-up visits, with BioFlx crowns showing the highest score for crown substance loss at the six-month follow-up, PZCs showing the highest score for crown debonding at 12 months, and SSCs showing the lowest [18].

Regarding BioFlx crown retention, the crowns in the present study showed excellent retention at both follow-up periods: all crowns remained intact at three months, and chipping or partial loss of material occurred in only one crown by six months after treatment. Our findings were notably more favorable than those reported by Rao et al. (2026), who found that 32% of BioFlx crowns dislodged after six months, although this difference was not statistically significant [19]. In contrast, Patil et al. (2024) observed a statistically significantly higher rate of BioFlx crown loss compared to SSCs [20]. These findings were attributed to the inability to crimp BioFlx crowns after trimming, which they believed contributed to retention failure, as BioFlx crowns are designed for a snug fit rather than a snap fit compared with SSCs.

Furthermore, Singh et al. (2025) compared the 12-month clinical performance of SSCs and BioFlx crowns in primary molars [21]. SSCs showed slightly higher crown retention (100% vs. 98%), marginal integrity (96% vs. 92%), and overall clinical success (95% vs. 92%), while gingival health was comparable between groups with no significant difference [21].

The crown staining outcomes observed in the present study were comparable to those reported by Rao et al. (2026), who reported that 94% of crowns at the six-month follow-up visit received a Bravo score, reflecting mild surface staining that remained amenable to polishing [19]. In the present study, 93% of crowns were clinically ideal, with no apparent staining, and received an Alpha score at the three-month follow-up visit, decreasing slightly to 91.8% at six months. The proportion of crowns receiving a Bravo score increased from 7% at three months to 8% at six-months. The remaining crowns exhibited only minimal discoloration, with no case of severe or irreversible staining recorded throughout the follow-up periods. Collectively, studies suggest that BioFlx crowns demonstrate clinically acceptable staining resistance over a short follow-up period, with any discoloration being superficial and manageable through routine prophylactic measures.

Crown surface integrity was largely preserved, with most crowns showing no changes or only minor indentations (dimples); however, a small number (4.9%) exhibited clinically unacceptable surface perforations reaching Charlie grade by six months, and only 22 (36.1%) reached Alpha score and were clinically ideal, with no changes to the crown.

The observed occlusal dimpling is consistent with the surface adaptation described by the manufacturer as a feature of the material’s self-adaptable behavior [22], and is possibly linked to bruxism, excessive occlusal loading, or individual masticatory variation (Figure 2). These findings are comparable with those of Rao et al. (2026), who reported a more pronounced decline in occlusal surface integrity at six months, with only 67.6% of crowns retaining an Alpha score, 29.4% progressing to Bravo, and 2.9% reaching a Charlie grade with tooth surface exposure [19]. The superior occlusal outcomes observed in the present study may reflect differences in patient characteristics or follow-up methodology; however, both studies collectively emphasize the need for extended observation periods and careful patient selection to fully assess the long-term occlusal durability of BioFlx crowns.

Goswami et al. (2024) reported favorable six-month retention and aesthetic outcomes in a case series of primary molars restored with BioFlx crowns, with high satisfaction among children and their parents [9]. These findings are consistent with the present study, which also demonstrated high crown retention and favorable immediate child and parental satisfaction. The authors additionally reported an occlusal dimple in one crown during follow-up, comparable to the clinically acceptable surface indentations frequently observed in the present study. However, unlike the limited descriptive findings of that three-case series, the present study also identified a small number of clinically unacceptable perforations [9].

In the present study, the clinical assessment of BioFlx crowns extended beyond retention and aesthetics to encompass occlusal harmony, opposing dentition wear, and the integrity of the crown surface material. Occlusal assessment revealed consistently ideal outcomes; no wear of the opposing dentition was detected throughout the follow-up periods, which may reflect the favorable biomechanical properties of the BioFlx material and its compatibility with the occlusal forces encountered in the primary dentition.

In addition, children and parents expressed high satisfaction with the aesthetic outcomes of BioFlx crowns, including color, shape, size, and overall appearance, in the present study, a finding that aligns with Rao A. et al. (2026), who reported that 82.4% of parents were satisfied with crown aesthetics at six months [19]. Aesthetic satisfaction, however, favored BioFlx crowns.

This study has several limitations that should be considered when interpreting the findings. First, the inclusion criteria resulted in a relatively selected cohort with favorable baseline occlusal conditions, which may have contributed to the positive outcomes observed under these relatively controlled clinical circumstances. Consequently, the clinical performance of BioFlx crowns may have been overestimated in the broader pediatric population. Second, the absence of a comparator group substantially limits interpretation of the results. As this was a single-arm study, the findings cannot establish whether BioFlx crowns are equivalent or superior to SSCs or PZCs. In addition, the observed improvements in plaque accumulation and gingival health cannot be attributed solely to the crown material, as they may also have been influenced by unmeasured factors, including improved oral-hygiene practices and repeated professional follow-up visits. In addition, 10 crowns were unavailable for evaluation at the six-month follow-up due to participant non-attendance, which may have introduced attrition bias. In addition, several children contributed more than one crown, and each crown was assessed repeatedly over time. Therefore, observations were not statistically independent. Because the study was exploratory and descriptive, no generalized estimating equation or mixed-effects model was performed; future adequately powered studies should account for clustering at the child level and repeated measurements over time. Although examiners underwent training and calibration sessions before data collection, formal intra- and inter-examiner reliability testing was not performed, and reliability coefficients, such as weighted kappa statistics, were not calculated. This should be considered when interpreting the clinical assessment outcomes.

Satisfaction was assessed only immediately after crown placement; therefore, these findings reflect immediate post-treatment satisfaction and do not provide evidence of satisfaction throughout the six-month follow-up period. The uniformly high satisfaction scores and limited variability may indicate a ceiling effect, potentially restricting the questionnaire’s ability to discriminate among participants with high satisfaction levels. In addition, satisfaction was assessed immediately after crown placement and may have been influenced by the novelty of the restoration or other short-term contextual factors. Furthermore, the retrospective registration of the study is an important reporting limitation, as the study design and outcomes were not publicly registered prior to participant enrollment. Finally, the satisfaction questionnaire was adapted from a previously validated instrument with minor modifications, though the adapted version itself was not re-validated.

The relatively small sample size and short follow-up period further limit the generalizability of the findings and the ability to assess longer-term outcomes. Therefore, larger, prospectively registered, longitudinal, comparative, controlled clinical studies involving more diverse pediatric populations under routine clinical conditions and with extended follow-up are warranted to support the clinical use of this new product, particularly to evaluate changes in gingival health and satisfaction over time.

## 5. Conclusions

Within the limitations of this study and the selected cohort, BioFlx crowns exhibited favorable short-term clinical performance in primary molars observed over a six-month period. However, material integrity was less favorable than the other evaluated parameters, as clinically acceptable surface indentations were frequently observed and a small number of crowns developed clinically unacceptable perforations. Children and parents also reported high immediate post-treatment satisfaction following crown placement.

## Figures and Tables

**Table 1:** Clinical parameters and scoring criteria, including plaque accumulation, bleeding on probing, and modified United States Public Health Service (USPHS) system criteria used for outcome assessment in the study.

**Plaque accumulation on the crown measured by Greene and Vermillion Simplified Oral Hygiene Index (OHI-S)**
0 No debris or stain present. 1 Soft debris covering not more than one-third of the tooth surface, or presence of extrinsic stains without other debris regardless of surface area covered. 2 Soft debris covering more than one-third, but not more than two-thirds, of the tooth surface 3 Soft debris covering more than two-thirds of the tooth surface
**Gingival health measured by Löe and Silness Gingival Index (GI)**
0 No bleeding 1 Bleeding point appears a few seconds after probing 2 Bleeding immediately after the probing 3 Profuse bleeding immediately after probing spreading towards the marginal gingiva
**Proximal contact (mesial and distal)**
Alpha—clinically ideal, with the contact area having acceptable resistance to the passage of floss Bravo—clinically acceptable, with the contact area too tight or loose to the passage of floss Charlie—clinically unacceptable, with no contact with the adjacent tooth
**Occlusion**
Alpha—clinically ideal, with the crown being in harmony with occlusion Bravo—clinically acceptable, with the crown slightly high or low in occlusion Charlie—clinically unacceptable, with the crown needing to be replaced
**Crown retention after cementation**
Alpha—good retention Bravo—crown partially dislodged or mobile Charlie—complete dislodgment of crown
**Staining of crown**
Alpha—clinically ideal, with no apparent staining Bravo—clinically acceptable, with staining that could be polished away Charlie—clinically unacceptable, with heavy staining that could not be polished away
**Wear of the opposing tooth**
Alpha—clinically ideal, with no evidence of wear Bravo—clinically acceptable, with mild wear of the opposing tooth Charlie—clinically unacceptable, with severe wear of the opposing tooth
**Marginal integrity**
Alpha—clinically ideal, with no evidence of ditching or gap along the gingival crown margin. Bravo—clinically acceptable, with evidence of ditching or gap at the gingival crown margin that does not extend to the depth of the preparation Charlie—clinically unacceptable, with the crown being mobile or evidence of a gap extending the entire depth of the preparation margin, making crown replacement necessary.
**Surface of the crown material**
Alpha—clinically ideal, with no changes to the crown Bravo—clinically acceptable, with indentation on the crown surface with no exposure of underlying cement nor tooth structure Charlie—clinically unacceptable requiring replacement, with perforation on the crown exposing underlying cement and/or tooth structure

**Table 2:** Demographic characteristics, dental history, and oral health status of the study participants (*n* = 28 children).

Demographics Characteristics	Categories	*N* (%)
Age in years	6–7 years	12 (42.9)
	8–9 years	16 (57.1)
Gender	Female	16 (57.1)
	Male	12 (42.9)
Type of school	Public	16 (57.1)
	Private	12 (42.9)
Number of siblings	No siblings	3 (10.7)
	One	9 (32.1)
	Two or more	16 (57.1)
Child order of birth	First	16 (57.1)
	Second or later	12 (42.9)
Mothers’ age in years	25–34	12 (42.9)
	35–50	16 (57.1)
Fathers’ age in years	30–39	15 (53.6)
	40–55	13 (46.4)
Mothers’ education level	High school or less	8 (28.6)
	College or higher	20 (71.4)
Fathers’ education level	High school or less	1 (3.6)
	College or higher	27 (96.4)
Mothers’ employment	Yes	18 (64.3)
	No	10 (35.7)
Fathers’ employment	Yes	28 (100.0)
	No	0 (0.0)
Average monthly family income	Low	0 (0.0)
	Low to middle	9 (32.1)
	Middle to high	19 (67.9)
Dental history	Yes	13 (46.4)
	No	15 (53.6)
Participant level Oral Health Status		
dmft/DMFT	1–5	5 (17.9)
	6–10	20 (71.4)
	11 or more	3 (10.7)
Oral hygiene measured by Greene and Vermillion Simplified Oral Hygiene Index (OHI-S)	0–0.6 = Good	1 (3.6)
	0.7–1.8 = Fair	15 (53.6)
	1.9–3 = Poor	12 (42.9)
Gingival health measured by Löe and Silness Gingival Index (GI)	0.1–1 = Mild gingivitis	27 (96.4)
	1.1–2.0 = Moderate Gingivitis	1 (3.6)
	2.1–3.0 = Severe Gingivitis	0

**Table 3:** The preoperative baseline clinical status of the included teeth (*n* = 71 teeth).

Teeth Status	Categories	*N* (%)
Arch	Maxillary	42 (59.2)
	Mandibular	29 (40.8)
Primary molar	First	41 (57.7)
	Second	30 (42.3)
Side	Right	34 (47.9)
	Left	37 (52.1)
Number of carious surfaces	Two surfaces	62 (87.3)
	Multi-surfaces	9 (12.7)
Pulp Status	Normal	65 (91.5)
	Previous pulpotomy	3 (4.2)
	Needed pulpotomy	3 (4.2)
Status of opposing tooth	Natural tooth	45 (63.4)
	Composite or Amalgam restoration	10 (14.1)
	Stainless-steel crown	12 (16.9)
	No opposing tooth	4 (5.6)
Status of adjacent tooth	Natural tooth	62 (87.3)
	Composite or Amalgam restoration	5 (7.0)
	Stainless-steel crown	3 (4.2)
	No adjacent tooth	1 (1.4)

**Table 4:** The oral hygiene, gingival health, and clinical parameters of the included teeth at the baseline, three-month, and six-month follow-up visits.

Plaque Accumulation on the Crown	Baseline *n* = 71 *N* (%)	Three-Month *n* = 71 *N* (%)	Six-Month *n* = 61 *N* (%)
0 = No debris or stain present.	Not applicable	47 (66.2)	20 (32.8)
1 = Soft debris covering not more than one-third of the tooth surface, or presence of extrinsic stains without other debris regardless of surface area covered.		17 (23.9)	30 (49.2)
2 = Soft debris covering more than one-third, but not more than two-thirds, of the tooth surface.		7 (9.9)	9 (14.8)
3 = Soft debris covering more than two-thirds of the tooth surface.		0 (0.0)	2 (3.3)
**Gingival health**			
0 = No bleeding.	Not applicable	69 (97.2)	58 (95.1)
1 = Bleeding point appears a few seconds after probing.		2 (2.8)	1 (1.6)
2 = Bleeding immediately after the probing.		0 (0.0)	2 (3.3)
3 = Profuse bleeding immediately after probing spreading towards the marginal gingiva.		0 (0.0)	0 (0.0)
**Proximal Contact Mesial**			
0 = Alpha—clinically ideal, with the contact area having acceptable resistance to the passage of floss.	66 (93.0)	66 (93.0)	57 (93.4)
1 = Bravo—clinically acceptable, with the contact area too tight or loose to the passage of floss.	4 (5.6)	4 (5.6)	3 (4.9)
2 = Charlie—clinically unacceptable, with no contact with the adjacent tooth.	1 (1.4)	1 (1.4)	1 (1.6)
**Proximal Contact Distal**			
0 = Alpha—clinically ideal, with the contact area having acceptable resistance to the passage of floss.	69 (97.2)	67 (94.4)	57 (93.4)
1 = Bravo—clinically acceptable, with the contact area too tight or loose to the passage of floss.	1 (1.4)	3 (4.2)	3 (4.9)
2 = Charlie—clinically unacceptable, with no contact with the adjacent tooth.	1 (1.4)	1 (1.4)	1 (1.6)
**Occlusion**			
0 = Alpha—clinically ideal, with the crown being in harmony with occlusion.		71 (100)	61 (100)
1 = Bravo—clinically acceptable, with the crown occluding slightly high or low in occlusion.		0 (0.0)	0 (0.0)
2 = Charlie—clinically unacceptable, with the crown needing to be replaced.		0 (0.0)	0 (0.0)
**Crown retention after cementation**			
0 = Alpha—good retention.		71 (100)	60 (98.4)
1 = Bravo—crown partially dislodged or mobile.		0 (0.0)	1 (1.6)
2 = Charlie—complete dislodgment of crown.		0 (0.0)	0 (0.0)
**Staining of crown**			
0 = Alpha—clinically ideal, with no apparent staining.		66 (93.0)	56 (91.8)
1 = Bravo—clinically acceptable, with staining that could be polished away.		5 (7.0)	5 (8.2)
2 = Charlie—clinically unacceptable, with heavy staining that could not be polished away.		0 (0.0)	0 (0.0)
**Wear of opposing tooth**			
0 = Alpha—clinically ideal, with no evidence of wear		71 (100)	61 (100)
1 = Bravo—clinically acceptable, with mild wear of the opposing tooth.		0 (0.0)	0 (0.0)
2 = Charlie—clinically unacceptable, with severe wear of the opposing tooth.		0 (0.0)	0 (0.0)
**Marginal integrity**			
0 = Alpha—clinically ideal, with no evidence of ditching or gap along the gingival crown margin.		67 (94.4)	57 (93.4)
1 = Bravo—clinically acceptable, with evidence of ditching or gap at the gingival crown margin that does not extend to the depth of the preparation.		4 (5.6)	4 (6.6)
2 = Charlie—clinically unacceptable, with the crown being mobile or evidence of a gap extending the entire depth of the preparation margin, making crown replacement necessary.		0 (0.0)	0 (0.0)
**Surface of the crown material**			
0 = Alpha—clinically ideal, with no changes to the crown.		30 (42.3)	22 (36.1)
1 = Bravo—clinically acceptable, with indentation on the crown surface with no exposure of underlying cement nor tooth structure		40 (56.3)	36 (59.0)
2 = Charlie—clinically unacceptable requiring replacement, with perforation on the crown exposing underlying cement and/or tooth structure		1 (1.4)	3 (4.9)

**Table 5:** Children and parental immediate post-treatment satisfaction with BioFlx crowns after placement (*n* = 28 children and parents).

Satisfaction Parameter	Child	Parent
How satisfied are you with the color of the crown?	9.7 ± 0.7	9.4 ± 1.1
How satisfied are you with the shape of the crown?	9.8 ± 0.4	9.7 ± 0.9
How satisfied are you with the size of the crown?	9.9 ± 0.3	9.8 ± 0.6
How would you rate your overall appearance satisfaction?	9.8 ± 0.4	9.5 ± 0.8

## Data Availability

The original contributions presented in this study are included in the article/Supplementary Material. Further inquiries can be directed to the corresponding author.

## Supplementary Materials

The following supporting information can be downloaded at: https://www.mdpi.com/article/10.3390/children13091258/s1, Supplementary File S1: Satisfaction questionnaire and scoring instructions; Supplementary File S2: Anonymized study dataset; Supplementary File S3: Ethical approval documentation; Supplementary File S4: Blank parental informed consent form; Supplementary File S5: Blank child assent form; and Supplementary File S6: Blank consent form for publication of anonymized clinical photographs.

## Abbreviations

The following abbreviations are used in this manuscript:

SSCs	Stainless-Steel Crowns
PZCs	Prefabricated primary zirconia crowns
Bis-GMA	Bisphenol A-glycidyl methacrylate
KAUFD	King Abdulaziz University Faculty of Dentistry
CONSORT	Consolidated Standards of Reporting Trials
ASA	American Society of Anesthesiologists
BWs	Bitewings radiographs
SR	Saudi Riyals
dmft	Decayed, Missing, Filled for Primary Teeth
DMFT	Decayed, Missing, Filled for Permanent Teeth
USPHS	United States Public Health Services
GI	Löe and Silness Gingival Index
OHI-S	Greene and Vermillion Simplified Oral Hygiene Index
