# Supplementary material for: Clinical Performance and Immediate Child and Parental Satisfaction with BioFlx Crowns in Primary Molars: A Prospective, Single-Arm, Non-Randomized Interventional Study
Source: Children (Basel). 2026 Sep 16;13(9):1258. doi: 10.3390/children13091258 (PMC13605739; doi:10.3390/children13091258)
Supplement: Supplementary file 1 [file children-13-01258-s001.zip › S1 Satisfaction questionnaire and scoring instructions.pdf]

BioFlx crown Satisfaction Survey

After cementation of the BioFlx crown, rate your satisfaction with the restoration. Please consider the crown's appearance, color, shape, size, and their overall satisfaction, and select the number on the 10-point scale that best represents your opinion.

1- How satisfied are you with the appearance of the crown?

1 2 3 4 5 6 7 8 9 10
Not satisfied at all
Very satisfied

2- How satisfied are you with the color of the crown?

1 2 3 4 5 6 7 8 9 10
Not satisfied at all
Very satisfied

3- How satisfied are you with the shape of the crown?

1 2 3 4 5 6 7 8 9 10
Not satisfied at all
Very satisfied

4- How satisfied are you with the size of the crown?

1 2 3 4 5 6 7 8 9 10
Not satisfied at all
Very satisfied

5- How would you rate your overall satisfaction?

1 2 3 4 5 6 7 8 9 10
Not satisfied at all
Very satisfied
