# Supplementary material for: Clinical Performance and Immediate Child and Parental Satisfaction with BioFlx Crowns in Primary Molars: A Prospective, Single-Arm, Non-Randomized Interventional Study
Source: Children (Basel). 2026 Sep 16;13(9):1258. doi: 10.3390/children13091258 (PMC13605739; doi:10.3390/children13091258)
Supplement: Supplementary file 1 [file children-13-01258-s001.zip › S3 ethical approval documentation.PDF]

**KINGDOM OF SAUDI ARABIA**<br>**Ministry of Higher Education**<br>**KING ABDULAZIZ UNIVERSITY**<br>**FACULTY OF DENTISTRY**		**المملكة العربية السعودية**<br>**وزارة التعليم العالي**<br>**جامعة الملك عبد العزيز**<br>**كلية طب الأسنان**

الرقم : ٤٥ ١٢٤٢٧١ التاريخ ٢ /٨٢/ 1445 :هـ المشفوعات................................:

Dr. Shahad Abudawood

Department: Department of Pediatric dentistry.
Subject: REC Decision
Proposal No.: 53-03-24

The committee has reviewed your proposal entitled:

"Evaluation of Clinical and Radiographic Performance, and Parental and Children's Satisfaction of BioFlx Crowns in Primary Molars: Randomized Clinical Trial: 24-Month Follow-up."

Please be advised that with respect to: (1) the rights and welfare of the individual (s) involved, (2) the appropriateness of the methods to be used to secure informed consent, and (3) the risks and potential benefits of the investigation, the Committee considers your project:

☐ Exempt
☒ Acceptable (E)
☐ Acceptable with reservation noted *(see attached letter)*
☐ not acceptable for reasons noted *(see attached letter)*

☐ Follow-up: The Committee wishes to have a status report on this project on / /

SIGNED FOR THE COMMITTEE BY:

Approval Date: 10/05/2024.

Rayan A. Kayal BDS, DSC
Vice Dean for Postgraduate Studies & Scientific Research
Director of Research Ethics Committee
Faculty of Dentistry, King Abdul Aziz University

****
****

Type Project	:	☒ NEW	☐ RENEWAL
Human Risk	:	☐ Yes	☒ No
Source of Support	:	☐ outside Funding	☒ Department/other
Agency (Potential)	:	Agency No.	

Are any of the following involved: ☒ Yes ☐ No
If yes, which category (ies) :

☒Minors ☐ Fetuses ☐ Abortuses ☐ Pregnant Women
☐ Prisoners ☐ Mentally Retarded ☐ Mentally Disabled

CC: Dean Department Chairman Follow-up (Book) File

جامعة الملك عبد العزيز كلية طب الأسنان KING ABDULAZIZ UNIVERSITY Faculty Of Dentistry
