# Supplementary material for: Clinical Performance and Immediate Child and Parental Satisfaction with BioFlx Crowns in Primary Molars: A Prospective, Single-Arm, Non-Randomized Interventional Study
Source: Children (Basel). 2026 Sep 16;13(9):1258. doi: 10.3390/children13091258 (PMC13605739; doi:10.3390/children13091258)
Supplement: Supplementary file 1 [file children-13-01258-s001.zip › S4 blank parental informed consent form.pdf]

RESEARCH ETHICS COMMITTEE
of the Faculty of Dentistry (REC-FD)
Consent Form

A- Title of Research
Evaluation of Clinical and Radiographic Performance, and Parental and Children’s Satisfaction of BioFlx Crowns in Primary Molars: Randomized Clinical Trial: 24-Month Follow-up

ا. عنوان البحث
دراسة لتقييم الأداء الإكلينيكي والإشعاعي ورضاء الوالدين وأطفالهم على تيجان البيوفلكس ذوالتغطية الكاملة لعلاج الأضراس الخلفية اللبنية: بحث إكلينيكي عشواءي مع مراجعة لمدة ٢٤ شهر.

Part I – Research Participant Information Sheet:

الجزء الأول – معلومات للمشارك في البحث:

B. Description of the Research:
This study aims to assess the clinical and radiographic performance of BioFlx crowns as a full-coverage restorative treatment option in primary molars in pediatric patients after 6, 12, 18, and 24 months of treatment. Also, parental and children's satisfaction will be evaluated immediately after placement and after six months of treatment.

ب. وصف البحث:
تهدف الدراسة لتقييم الأداء الإكلينيكي والإشعاعي ورضاء الوالدين وأطفالهم على تيجان البيوفلكس ذو التغطية الكاملة لعلاج الأضراس الخلفية اللبنية وسوف يتم التقييم مباشرة بعد العلاج وبعد ستة أشهر بعد العلاج.

C. Potential Risks and Discomforts:
Regular risks associated with crown preparation, with no extra risks, are associated, such as but not limited to bleeding, pain and discomfort and crown dislodgment.

جـ. المضاعفات المحتملة:
المخاطر المعتادة المرتبطة بتحضير التاج، دون وجود مخاطر إضافية. على سبيل المثال لا الحصر: النزيف والألم والانزعاج وخسارة التاج.

D. Potential Benefits:
Participants will benefit from a new esthetic restorative option.

د. الفوائد المحتملة:
سيستفيد المشاركون من خيار علاجي جمالي.

E. Cost/s:
Participants in the research will bear no additional costs.

هـ. التكاليف / التعويضات المالية:
لن يتم مطالبة المشارك في البحث بأي تكاليف إضافية.

F. Compensation / Treatment:
No risks are associated with this study. In the event of injury resulting from participation in this research study, the researcher will provide you with the needed treatment, facilities, and professional attention.

و. التعويضات / العلاج:
في حالة حدوث أي ضرر لا قدر الله جراء مشاركتك بهذه الدراسة سيتم تقديم العناية الطبية اللازمة.

G. Voluntary Participation:
Participation in this study is voluntary. Withdrawal from this research study will not affect your ability to receive alternative methods of dental care available at KAUFD.

ز. المشاركة الطوعية:
المشاركة في هذه الدراسة طوعية وإذا قررت عدم المشاركة فإنك لن تتعرض لأي مضايقات، كما أن قرارك بالانسحاب من الدراسة لن يؤثر على تلقيك لخدمة علاجية بديلة في كلية طب الأسنان.

H. Confidentiality:
Your identity as a participant in this research study will remain confidential concerning any publications that may result from this study. Furthermore, your Dental/medical record may be reviewed by the Research Advisory Council or the agency sponsoring this research in accordance with applicable laws and regulations.

ح. السرية:
كمشارك في هذه الدراسة ستكون هويتك سرية في جميع المنشورات، المتعلقة بنتائج الدراسة، ويمكن الاطلاع عليه من قبل المجلس الاستشاري للأبحاث أو الجهة الداعمة للدراسة في حدود النظم والقوانين المطبقة بهذا الخصوص

I. Contact Person(s):
Dr. Shahad Abudawood/ Dr. Sara M. Bagher
0503611195 / 0547433525

ط. الأشخاص الذين يمكن الاتصال بهم:
د. سارة باغر/ د. شهد أبو داود
0547433525/0503611195

A signed copy of the consent form will be given to you.

سيتم تزويدك بنسخة موقعة من هذا الإقرار.

PART II - Authorization for performance of certain procedures:

الجزء الثاني- تفويض باستعمال إجراء طبي:

RESEARCH ETHICS COMMITTEE
of the Faculty of Dentistry (REC-FD)
Consent Form

1-I/surrogate authorize Dr. Ahmed Essam Mohammed /Dr. Lolo Ahmed Almansour and their associates participated in this research using the data that was given in the interview questionnaire.

١. بهذا أفوض (المشاركين) الدكتور/ة: احمد عصام محمد، لولو احمد المنصور - أو أحد المشاركين معهم بالمشاركة في البحث - خلال السماح باستخدام البيانات المعطاة في الاستبانة.

2. I acknowledge that I have read or had explained to me in a language I understand, the attached Research Participant Information sheet, and that Dr. Ahmed Essam Mohammed /Dr. Lolo Ahmed Almansour has explained to me the nature of the research, any benefits to be expected, the attendant discomforts and risks reasonably to be expected, and the possibility of complications.

٢. أقر بأنني قد قرأت - أو قد شرحت لي بلغة أفهمها - جميع المعلومات المتعلّقة بالمشاركة بالبحث والمرفقة، وأن الدكتور/ة: احمد عصام محمد، لولو احمد المنصور أو أحد المشاركين معهم بالمشاركة في البحث - قد أوضحوا لي الغرض من البحث والفوائد المرجوة والمخاطر والمضاعفات المتوقع حدوثها

3. I understand that I am not entitled to reimbursement for expenses incurred due to my participation in this study.

٣. من المفهوم لديّ بأنني لا يجب على استرداد المصروفات التي نتجت عن مشاركتي في هذه الدراسة.

4. I understand that I am free not to answer the interview or to refuse it at any time. Also, I understand that such refusal will not affect my ability to receive any dental/medical care made necessary.

٤. وأفهم أن لي مطلق الحرية بعدم تعبئة كل البيانات أو رفض المقابلات الهاتفية. كما أفهم بأن انسحابي من هذه الدراسة لن يؤثر على حقي في تلقّي العناية الطبية اللازمة.

5. I confirm that I have read, or had read to me, the foregoing authorization and that all blanks or statements requiring completion were properly completed before I signed.

٥. بهذا أؤكد بأنني قد قرأت (أو قرأ لي) هذا التفويض وأن جميع المعلومات اللازمة قد تمت تعبئتها بدقة قبل توقيعي عليه

Patient name: **اسم المريض:**

File #: **رقم الملف في كلية طب الأسنان:**

Patient/Surrogate: **اسم ولي الأمر لو كان المريض غير مكلف:**

Relationship: **صلة القرابة:**

Signature: **Date:** **التاريخ:** **التوقيع:**

6. I confirm that I have accurately translated and/or read the information to the subject or his/her surrogate.

٦.أؤكد بأنني قد قرأت/ شرحت جميع المعلومات المذكورة بدقة للمريض . أو لولي أمره.

Name: **الاسم:**

Signature: **التوقيع:**

KAUFD ID #: **رقم الملف في كلية طب الأسنان:**

Date: **التاريخ:**

7.I have fully explained to the above patient/ relative/ guardian the nature and purpose of the Research and the reasonable benefits to be expected.

٧. أقر بأنني قد شرحت بصورة كاملة للمريض / أو قريبه (ولي أمره) طبيعة والغرض من هذا الإجراء. كما أنني قد أوضحت النتائج أو المخاطر المختلفة.

RESEARCH ETHICS COMMITTEE
of the Faculty of Dentistry (REC-FD)
Consent Form

I understand that the above patient/ relative/ guardian understands the nature, purposes, benefits, and risks participation in this research before signing of this informed consent.

I have also offered to answer any questions the above patient/ relative/ guardian might have with respect to such procedures and have fully and completely answered all such questions.

Signature of Principal Investigator/ Delegate):
Dr. Shahad Abudawood

Print Name: Dr. Shahad Abudawood
Title: Assistant Professor
Date: 28/1/2024

This Consent Document is approved for use by the
Research Ethics Committee of KAUFD
REC # : ________________________________

Date From: ______________________________

To:_____________________________________

من المفهوم لديّ بأن المريض المذكور أعلاه (أو قريبه أو ولي أمره) قد فهم طبيعة الدراسة والهدف منها والفوائد والمخاطر المترتبة على المشاركة فيها قبل توقيعه على الموافقة بالمشاركة.

وقد قمت بتوضيح استعدادي للإجابة على جميع أسئلة المريض/ قريبه/ ولي أمره بالإجابة على تلك المتعلقة بالإجراء موضوع الدراسة وقمت فعلاً بالإجابة كاملةً على جميع الأسئلة بطريقة واضحة ومرضية.

توقيع الباحث الرئيسي أو من ينوب عنه:

الاسم: د. شهد أبو داود
الوظيفة: أستاذ مساعد
التاريخ: ٢٠٢٤/١/٢٨

إقرار بالموافقة على المشاركة في الأبحاث
التي تتطلّب استعمال دواء/جهاز/ أو إجراءات خاصة
