# Supplementary material for: Clinical Performance and Immediate Child and Parental Satisfaction with BioFlx Crowns in Primary Molars: A Prospective, Single-Arm, Non-Randomized Interventional Study
Source: Children (Basel). 2026 Sep 16;13(9):1258. doi: 10.3390/children13091258 (PMC13605739; doi:10.3390/children13091258)
Supplement: Supplementary file 1 [file children-13-01258-s001.zip › S5 blank child assent form.pdf]

RESEARCH ETHICS COMMITTEE of
the Faculty of Dentistry (REC-FD)

Child Assent Form

أنا اعلم أن والديني (أبي أو أمي) وافقوا أن أشارك في هذا البحث عن تقييم الأداء الاكلينيكي والإشعاعي و مقارنة رضاء الوالدين وأطفالهم على تيجان البيوفلكس ذو التغطية الكاملة لعلاج الأضراس الخلفية اللبنية وسوف يتم التقييم مباشرة بعد العلاج وبعد ٦،١٢،١٨،٢٤ شهر بعد العلاج إذا كنت أنا أرغب بذلك.

أنا اعلم أنني أستطيع إيقاف هذا التقييم في أي وقت اريده، وسيكون الأمر على ما يرام إذا أردت التوقف.

الاسم: ____

التاريخ: ____

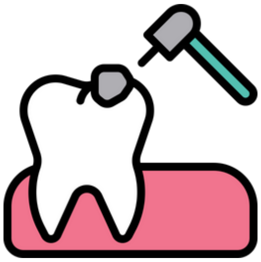

أولاً: سيتم تنويم السن ومن ثم ازالة التسوس باستخدام الفرشة المائية.

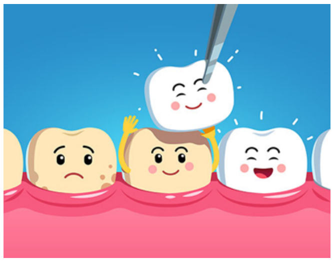

ثانياً: سيتم تجربة قبعة السن الابيض الجديد.

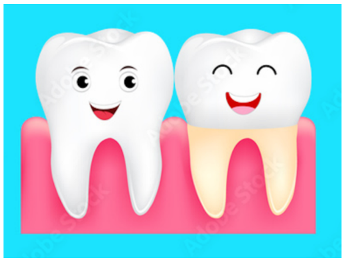

ثالثاً: سيتم الصاق القبعة البيضاء الجديدة حتى يظهر السن بشكل جميل.

RESEARCH ETHICS COMMITTEE of
the Faculty of Dentistry (REC-FD)

Child Assent Form

I have been told that my parents (mother or father) have said it's okay (or have given permission) for me to participate, if I want to, in this research about "assess the clinical and radiographic performance of BioFlx crowns as a full-coverage restorative treatment option in primary molars, and parental and children's satisfaction will be evaluated immediately after placement and up to 24 months of treatment".
I can stop using this assessment at whenever I want to, and it will be okay if I stop.

Name: ____

Date: ____

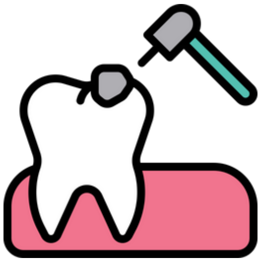

First: We will make the tooth sleep then the tooth decay will be removed by using water brush.

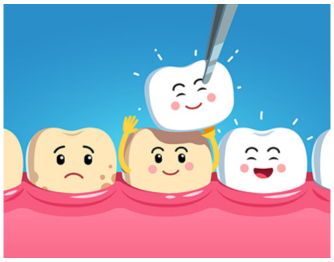

Second: We will try the new white hat.

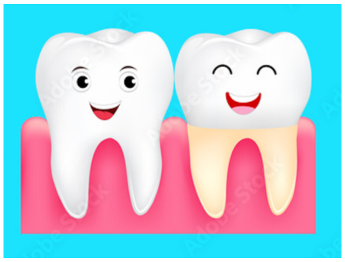

Third: We will stick the new white hat so the tooth will be great.

This Consent Document is approved for use
by the Research Ethics Committee of KAUFD

REC # : ________________________________

Date From: ________________________
To:________________________________

إقرار بالموافقة على المشاركة في الأبحاث
التي تتطلّب استعمال دواء/جهاز/ أو إجراءات خاصة

رقم الموافقة: ________________________________
التاريخ: من: ________________________________
إلى: ________________________________
