# Supplementary material for: Clinical Performance and Immediate Child and Parental Satisfaction with BioFlx Crowns in Primary Molars: A Prospective, Single-Arm, Non-Randomized Interventional Study
Source: Children (Basel). 2026 Sep 16;13(9):1258. doi: 10.3390/children13091258 (PMC13605739; doi:10.3390/children13091258)
Supplement: Supplementary file 1 [file children-13-01258-s001.zip › S6 blank consent form for publication of anonymized clinical photographs..pdf]

Consent Form

- **Title of Research**

Evaluation of Clinical and Radiographic Performance, and Parental and Children's Satisfaction of BioFlx Crowns in Primary Molars: Randomized Clinical Trial: 24-Month Follow-up

- **Consent for Dental Photography and Publication**

I, the undersigned parent/legal guardian, confirm that I have been informed that photographs of my child's teeth and oral cavity may be taken as part of the above-mentioned research study.

I understand and agree that:

- Dental photographs may be taken for research documentation and evaluation of the treatment outcome.
- The photographs may be used in scientific presentations, academic research, and publication in a scientific/medical journal.
- Reasonable measures will be taken to protect my child's privacy and confidentiality. No identifying information such as the child's name, medical record number, or face will be published with the photographs.
- I understand that once the photographs have been published, they may be accessible to readers of the scientific publication and may not be possible to completely withdraw them.
- I understand that refusing permission for photography or publication will not affect my child's dental care or participation in treatment.
- I have had the opportunity to ask questions, and my questions have been answered satisfactorily.

By signing below, I voluntarily give permission for my child's dental photographs to be taken and used for the purposes described above.

Patient name:

File #:

Parnets name:

Relationship:

Segnature:

Date:

- **عنوان البحث**

تقييم الأداء السريري والإشعاعي ورضا الوالدين والأطفال عن تيجان البيوفلكس ذوالتغطية الكاملة لعلاج الأضراس الخلفية اللبنية. بحث إكلينيكي عشوائي مع مراجعة لمدة ٢٤ شهر.

- **الموافقة على تصوير الأسنان والنشر العلمي**

انا ولي أمر/الوصي القانوني للطفل المذكور أدناه، أقر بأنني قد أُبلغت بأنه قد يتم تصوير أسنان وفم طفلي كجزء من البحث العلمي المذكور أعلاه.

أفهم وأوافق على ما يلي:

- قد يتم تصوير أسنان وفم طفلي لأغراض التوثيق والتقييم البحثي لنتائج العلاج.
- قد تُستخدم الصور في العروض العلمية والأبحاث الأكاديمية والنشر في مجلة علمية/طبية.
- سيتم اتخاذ الإجراءات المناسبة للحفاظ على خصوصية وسرية معلومات طفلي، ولن يتم نشر اسم الطفل أو رقم الملف الطبي أو صورة الوجه أو أي معلومات تعريفية مع الصور.
- أفهم أنه بعد نشر الصور في مجلة علمية، قد تصبح متاحة لقراءة المنشور العلمي، وقد لا يكون من الممكن سحبها بشكل كامل.
- أفهم أن رفض السماح بالتصوير أو النشر لن يؤثر على الرعاية السنية أو العلاج المقدم لطفلي.
- أتيحت لي الفرصة لطرح الأسئلة وتمت الإجابة عن استفساراتي بشكل واضح.

بتوقيعي أدناه، أوافق طوعًا على تصوير أسنان طفلي واستخدام الصور للأغراض الموضحة أعلاه.

اسم المريض:

رقم الملف في كلية طب الاسنان:

اسم ولي الامر اذا كان المريض غير مكلف:

صلة القرابة:

التوقيع:

التاريخ:
